# TCGADownloadHelper: simplifying TCGA data extraction and preprocessing

**DOI:** 10.3389/fgene.2025.1569290

**Published:** 2025-05-02

**Authors:** Alexandra Anke Baumann, Olaf Wolkenhauer, Markus Wolfien

**Affiliations:** ^1^ Department of Systems Biology and Bioinformatics, University of Rostock, Rostock, Germany; ^2^ Faculty of Medicine Carl Gustav Carus, Institute for Medical Informatics and Biometry, TUD Dresden University of Technology, Dresden, Germany; ^3^ Leibniz-Institute for Food Systems Biology at the Technical University of Munich, Freising, Germany; ^4^ Wallenberg Research Centre, Stellenbosch Institute of Advanced Study, Stellenbosch University, Stellenbosch, South Africa; ^5^ Center for Scalable Data Analytics and Artificial Intelligence, Dresden, Germany

**Keywords:** the cancer genome atlas (TCGA), sample preprocessing, Jupyter Notebook, lung cancer, genomic data commons (GDC) portal

## Abstract

The Cancer Genome Atlas (TCGA) provides comprehensive genomic data across various cancer types. However, complex file naming conventions and the necessity of linking disparate data types to individual case IDs can be challenging for first-time users. While other tools have been introduced to facilitate TCGA data handling, they lack a straightforward combination of all required steps. To address this, we developed a streamlined pipeline using the Genomic Data Commons (GDC) portal’s cart system for file selection and the GDC Data Transfer Tool for data downloads. We use the Sample Sheet provided by the GDC portal to replace the default 36-character opaque file IDs and filenames with human-readable case IDs. We developed a pipeline integrating customizable Python scripts in a Jupyter Notebook and a Snakemake pipeline for ID mapping along with automating data preprocessing tasks (https://github.com/alex-baumann-ur/TCGADownloadHelper). Our pipeline simplifies the data download process by modifying manifest files to focus on specific subsets, facilitating the handling of multimodal data sets related to single patients. The pipeline essentially reduced the effort required to preprocess data. Overall, this pipeline enables researchers to efficiently navigate the complexities of TCGA data extraction and preprocessing. By establishing a clear step-by-step approach, we provide a streamlined methodology that minimizes errors, enhances data usability, and supports the broader utilization of TCGA data in cancer research. It is particularly beneficial for researchers new to genomic data analysis, offering them a practical framework prior to conducting their TCGA studies.

## 1 Introduction

The rise of high-throughput sequencing data demands increased efforts to synchronize and organize this vast amount of information. Large repositories and databases have become essential for data sharing and collaboration among researchers from various fields. This is especially true for multi-omics data in oncology, which holds immense potential for advancing our understanding of cancer. One notable resource is The Cancer Genome Atlas (TCGA, https://portal.gdc.cancer.gov), which provides a comprehensive collection of cancer genomic data across diverse cancer types. TCGA offers various file formats, such as variant calling files (VCF, raw or annotated) from whole-exome or whole-genome sequencing, RNA sequencing count files, and even imaging data. Another valuable platform is cBioPortal, which facilitates the visualization of TCGA studies and additional cancer genomic data (Cerami et al., 2012; de Bruijn et al., 2023). However, while using pre-processed data is useful, researchers often require access to the raw data for more in-depth analyses. The Genomic Data Commons (GDC) Data Portal (Grossman et al., 2016) was developed to harmonize genomic data, integrating not only TCGA data but also datasets from other sources, such as the Therapeutically Applicable Research to Generate Effective Treatments (TARGET) or the Foundation Medicine Adult Cancer Clinical Dataset (FM-AD). The GDC Data Portal provides an API that can be used to search for these datasets, including metadata, and download them. However, using the GDC Data Transfer Tool (https://gdc.cancer.gov/access-data/gdc-data-transfer-tool) is the default method for downloading larger datasets.

Adding more complexity to the download process, multi-omics analyses require a multitude of diverse data. Whether for small-scale or large-scale studies, data transfer processes should remain simple to ensure that the primary focus stays on data analysis, which is of utmost importance. However, first-time users often find handling TCGA data challenging, as another tool, the GDC Data Transfer Tool, is required to download data from the TCGA portal. Each file is saved with a unique 36-character prefix in separate folders with other individual 36-character IDs, making it difficult to organize and correlate files with specific cases. Researchers can only map these files to the corresponding case IDs by utilizing the provided *Sample Sheet*. To facilitate the analysis of multi-modal data for each patient (e.g., integrating RNA and DNA data), it would be more intuitive to have the case ID as the file prefix.

To simplify the handling of TCGA data, the Waldron Lab developed TCGAutils (https://github.com/waldronlab/TCGAutils), which includes functionalities like mapping the case ID to the file name. However, while useful, these features are still fragmented, and a comprehensive, one-touch solution remains unavailable. Interactive computing environments, such as Jupyter Notebooks (Granger and Pérez, 2021), offer an ideal platform for combining code with narrative explanations. The interactive nature of these environments, along with their capability to support multiple programming languages, such as Python, R, and shell commands, enhances their usability for researchers. Snakemake (Mölder et al., 2021), a workflow management system, is another tool that enables researchers to combine diverse analysis steps within a single framework. A key advantage of Snakemake is its usage of sample names for each workflow step. Additionally, Snakemake supports the use of both shell commands and Python code in its rules. Thus, an integration of diverse supplementary tools is possible.

Here, we present a pipeline integrated within a Jupyter Notebook as well as a Snakemake pipeline that facilitate the download of TCGA data and subsequent patient-level data analysis. The primary goal of this pipeline is to simplify data download processes while reorganizing the file and folder structure to enhance usability and efficiency.

## 2 Methods

### 2.1 Sample data

Exemplarily, the data used in this project were sourced from the Lung Adenocarcinoma cohort [TCGA-LUAD (Collisson et al., 2014)] and the Lung Squamous Cell Carcinoma cohort [TCGA-LUSC (Hammerman et al., 2012)] from The Cancer Genome Atlas [TCGA (Weinstein et al., 2013)]. The TCGA Repository contains a wide array of data generated from various experimental strategies, each associated with specific data types. GDC provides a detailed documentation of the bioinformatic pipelines used with information on included tools (https://docs.gdc.cancer.gov/Data/Bioinformatics_Pipelines/DNA_Seq_Variant_Calling_Pipeline/).

In this study, the focus was on whole-genome sequencing (WGS) data, which included sequence alignment files in BAM format (aligned using BWA), copy number variation (CNV) data generated by AscatNGS, and variant calling format (VCF) files containing simple nucleotide variations (called by CaVEMan), insertions and deletions (detected by Pindel), and somatic structural variations (detected by BRASS). Furthermore, whole-exome sequencing (WES) data were utilized, including sequence alignment files in BAM format (aligned using BWA) and raw VCF files of somatic variants (called by MuSE, MuTect2, VarScan2, and Pindel) as well as further processed versions containing annotations. Genotyping array data were also downloaded, including allele-specific and gene-level CNV files generated by ASCAT2, ASCAT3, ABSOLUTE LiftOver, and DNAcopy. RNA sequencing (RNA-seq) data were employed as well, which included sequence alignment files in BAM format (aligned using STAR), gene expression quantification (STAR), splice junction quantification (STAR), and transcript fusion detection files (generated by STAR and Arriba). Additional data types available from TCGA included DNA methylation data, microRNA sequencing (miRNA-seq) data, comprising sequence alignment files in BAM format, miRNA expression quantification, and isoform miRNA expression quantification. ATAC-seq (Assay for Transposase-Accessible Chromatin) data, imaging files (diagnostic and tissue slides), and reverse-phase protein array data were also accessible, but have not been utilized during this underlying study.

### 2.2 Prerequisites and install

#### 2.2.1 Pipeline code and documentation availability

The source code and documentation of the *TCGADownloadHelper* are publicly available on GitHub: https://github.com/alex-baumann-ur/TCGADownloadHelper.

Download the GitHub repository, which contains a Jupyter Notebook, a Snakemake pipeline, necessary scripts, and a yaml file for conda environment setup. The code was written with Python (version 3.11.8) and Snakemake (version 7.32.4).

#### 2.2.2 Conda environments and packages

To use the sample download and preprocessing script as a one-touch pipeline within a Jupyter Notebook or as a Snakemake pipeline, one must first create a conda environment that includes the required packages. In particular, *Jupyter* (Granger and Pérez, 2021 (v1.0.0)), *gdc-client* (https://github.com/NCI-GDC/gdc-client (v2.3)), *snakemake* (Mölder et al., 2021 (v8.25.3)) *pandas* (https://pandas.pydata.org/pandas-docs/version/2.2.2/index.html, v2.0.3) and *pyyaml* (https://anaconda.org/conda-forge/pyyaml/, v6.0.1) are required. A yaml file containing the relevant conda packages is provided in our GitHub repository for easy environment setup. A conda environment can be set up with the following command:

conda env create −−name TCGAHelper −f envs/TCGADownloadHelper_env.yaml

The conda environment can be activated with the following command:

conda activate TCGAHelper

#### 2.2.3 Folder structure

**FIGURE 1:**
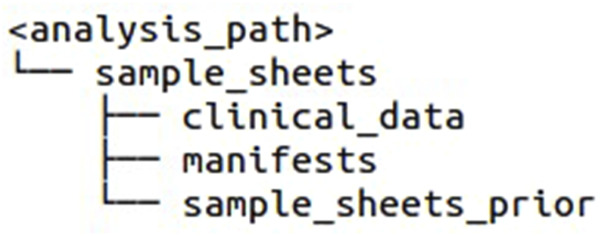
Folder structure for the sample sheets and manifest files, created by the pipeline.

In the local analysis folder, a sample_sheets folder with the following subdirectories is created by the script: clinical_data, manifests, and sample_sheets_prior (see Figure 1). After selecting the files to download from the TCGA Repository, the cart file (manifest file), sample sheet, and clinical metadata files must be saved in the appropriate subdirectories.

An optional feature allows users to download restricted access TCGA data using an access token. For that, log in to the GDC Data Portal with an NIH account, download an access token, and save it as a secured file.

### 2.3 Design and implementation

#### 2.3.1 Configuration file

The configuration file data/config.yaml contains all the necessary information for executing the *TCGADownloadHelper* pipeline to download TCGA data from a specified manifest file and sample sheet. Here, the locations of directories and file names have to be specified. For example, for restricted access files, an access token is required, of which the location must be specified in there. More detailed instructions on how to correctly fill out the configuration file are included within the file itself. This is the primary file that requires modification; no other adjustments are needed except for potential changes in the optional sample analysis Snakemake pipeline rules (Snakefile_sample_analysis).

#### 2.3.2 Pipeline steps

Our developed pipeline provides a step-by-step solution for improved TCGA data handling. All main TCGA data download and preprocessing steps are written in Python and are located in the scripts_TCGA_pipeline folder. The first step is to check the validity of configuration file entries, ensuring that all files listed in the configuration file exist. Afterward, the script merges the manifest(s) and sample sheet to map the case ID to the unique file ID. The script provides new names of the files where the matching case ID is put before the file suffix. If desired, a filter for specific case IDs from a previous analysis can be used. Furthermore, already downloaded files will be ignored and filtered out in the resulting adapted and filtered manifest file for the GDC-client download. Next, the script proceeds to download the TCGA data specified in the manifest(s) from previous steps or from the configuration file, placing the downloaded files in the folder <analysis_path>/00_raw_data. Once downloaded, symbolic links of the files are created in the folder <analysis_path>/01_sample_data with adapted names using the format <case_id>.<file_suffix>. The renamed files are sorted into folders, one for each analysis.

The pipeline can be executed as a Jupyter Notebook or a Snakemake pipeline after activating the TCGAHelper conda environment. The Jupyter Notebook is intuitive and can be run cell by cell in a browser, which opens with the following command:

jupyter notebook

The Snakemake pipeline can be run on the command-line as a one-touch pipeline. The pipeline starts with the following command after specifying the amount of cores to use:

snakemake −−cores <cores>

After preparing the data with those steps, a Sample Analysis Snakemake pipeline can be run to perform further analyses of all the downloaded data at once, if desired (see the following section).

#### 2.3.3 Sample analysis Snakemake pipeline

The pipeline provided is mainly intended as a data handling setup. However, we are providing a prototype Snakemake pipeline for further analysis of TCGA samples by case ID. Once one has decided on the specific analyses one wishes to perform, the corresponding rules can be defined within the Snakefile_sample_analysis file or rules of other pipelines can be reused. The pipeline can be either run within the Jupyter Notebook after setting the *snakemake_sample_analysis* parameter in the configuration file to “True” or separately on the command line with the following command:

snakemake −s Snakefile_sample_analysis −−cores <cores>

### 2.4 Hardware and software requirements

The *TCGADownloadHelper* (https://github.com/alex-baumann-ur/TCGADownloadHelper) was applied on a computer with a 12th Gen Intel^®^ CoreTM i7-1255U processor, 16 GB RAM, Linux Ubuntu 22.04.4 64-bit system, as well as on a server client with an Intel^®^ Xeon^®^ CPU E5-2630 v3 @ 2.40 GHz processor, 94.3 GB RAM, Linux Debian 6.7.12-1 (2024-04-24) 64-bit system. Both utilized conda version 23.7.4 and Python version 3.12.3. The pipeline is currently operational on unix operating systems.

## 3 Results

Our *TCGADownloadHelper* pipeline was designed to accomplish a simplified download of TCGA data, as well as a step to automatically rename the files by incorporating the sample sheet.

### 3.1 Obtaining TCGA manifest from GDC portal

The initial steps on the GDC Data Portal remain the same. To use the GDC Data Portal, follow these steps:.

**FIGURE 2:**
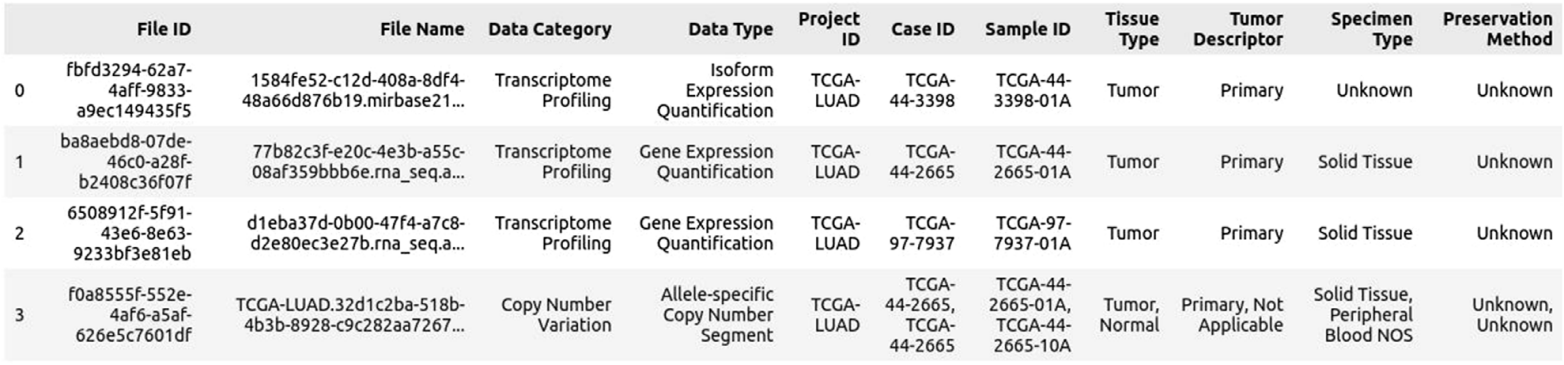
Example Sample sheet of lung cancer TCGA data.

**FIGURE 3:**
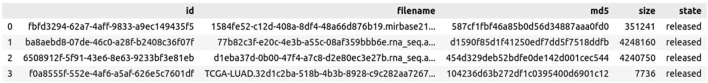
Example Manifest file of lung cancer TCGA data.

1. Navigate to the GDC Data Portal and click on the “Repository” tab. Depending on your needs, you can filter the data by files or by cases. Once filtered, select the files of interest or add all relevant data to the cart. To review your selection, click on the cart symbol at the top of the page.
2. Click on “Download Associated Data” and download the following files: Sample Sheet, Metadata, and (optional) Clinical TSV data, if additional information is required. The download of the sample sheet is most important, as it connects the file ID to the case ID (see Figure 2).
3. Click on “Download Cart” to download the manifest file (see Figure 3), which will be important for the gdc-client tool. These steps should be repeated for each new analysis, particularly when new data aspects or file types must be included.

### 3.2 Typical process and outcome without our pipeline

Commonly, to download data, an installation of the GDC Data Transfer Tool (graphical user interface or command line tool) is necessary (via a binary distribution or anaconda). The manifest file created in the previous step will be used to download the data. The graphical user interface has a drag and drop field for the manifest file.

The command line GDC Data Transfer Tool also needs the input of the manifest file location, as well as the optional user access token.

gdc−client download −m manifest.txt (−t user−token.txt)

**FIGURE 4:**
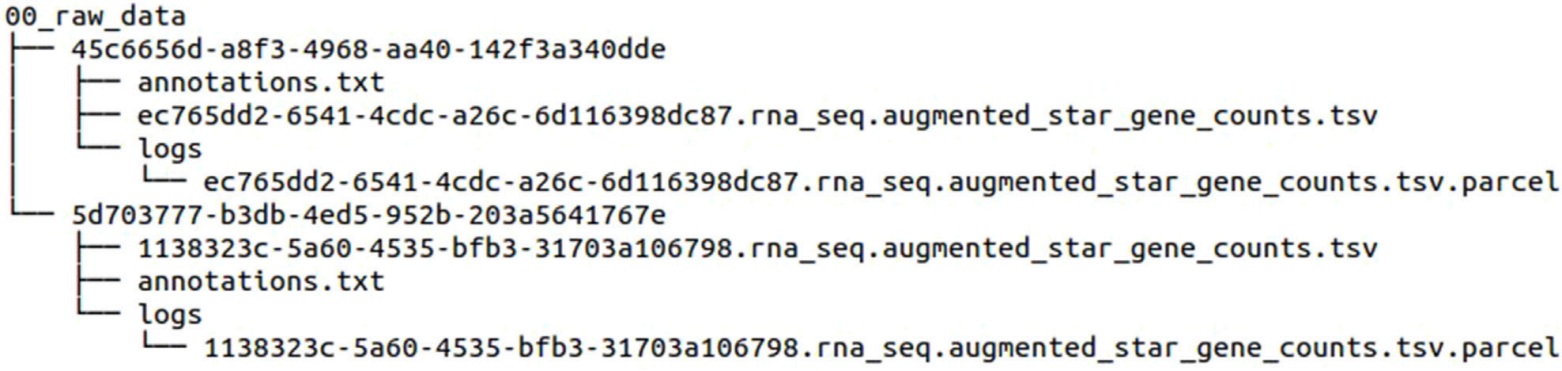
Folder structure and file names of the downloaded data files before using our pipeline.

After downloading the data, each file is stored in a separate folder with a unique 36-character ID. The final folder structure and file naming are as follows in Figure 4.

It is difficult to determine the contents of each folder and no mapping to the case ID has been conducted. Additionally, previously downloaded files will be downloaded again. If there are files that should not be included, each file must either be added separately to the cart at the TCGA Repository or downloaded unnecessarily. In general, further analyses are made more challenging when case IDs are required.

### 3.3 Process and outcome with the TCGADownloadHelper pipeline

**FIGURE 5:**
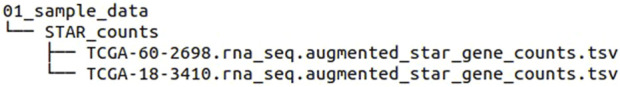
Folder structure and file names of the downloaded data files after using our pipeline.

After downloading the initial manifest file and adapting the required parameters in the *TCGADownloadHelper* configuration file, the Jupyter notebook or the Snakemake pipeline can be run. The resulting folder structure of the downloaded TCGA files is more human-readable and includes additional information about the specific Case IDs (see Figure 5).

Importantly, the raw files remain untouched and available, as only symbolic links (symlinks) are created in a separate folder. After that, a sample analysis Snakemake pipeline can be started within the Jupyter Notebook or separately to analyze all selected files, also by Case ID. This approach allows for the integration of multiple data types from the same patient in a single analysis.

Supplementary Table S1 compares the manual procedure with the use of the TCGADownloadHelper.

### 3.4 How to adapt and individualize the pipeline for new data types

Each TCGA download and data set is different from each other. While the goal is to apply the pipeline uniformly across all cases without changes, that is often not feasible in real-world applications. Therefore, minor adaptations might be necessary for customization. For instance, the config.yaml file allows for easy customization, such as incorporating additional analysis methods in the methods dictionary (methods_dict or including a sample sheet with case IDs to filter for.

#### 3.4.1 Extending the script for specific examples

One may also need to adapt the script, as file naming conventions can vary between datasets. Since standardizing renaming rules is not feasible, one can modify the additional Python script scripts_TCGA_pipeline/exceptions_renaming.py to further define how files are named in the data set at hand.

#### 3.4.2 Modifying rules in Snakemake

With some examples already included, adapting or adding new rules in the Snakefile_sample_analysis file becomes already streamlined. One primarily needs to adjust the suffixes of the input and output files. For convenience, each rule has its own Python script located in a separate scripts_snakemake folder, where the analysis tasks are defined and can be easily customized.

## 4 Discussion

Our developed *TCGADownloadHelper* pipeline simplifies the process of downloading and handling TCGA data by combining multiple steps into a streamlined workflow. It is more user-friendly and reduces the complexity associated with navigating the GDC-specific naming conventions. A major advantage of the pipeline is its ability to filter for relevant files or cases, a task that is otherwise challenging on the TCGA repository platform. Furthermore, it avoids unnecessary duplication of files by creating symbolic links for already downloaded files, thereby optimizing data storage and minimizing redundancy. By keeping the original files intact, the script ensures data traceability and provenance, which are crucial for reproducibility in scientific research. Existing tools like TCGAutils and curatedTCGAData streamline TCGA data handling within R/Bioconductor, offering helpful functions and ready-to-analyze datasets but often involve manual steps and limited scalability (Ramos et al., 2020). These limitations can hinder automation and reproducibility, especially for users lacking programming expertise or operating outside R environments. In contrast, our Python- and Snakemake-based pipeline enables easier, automated downloading and renaming of TCGA data, requiring only the TCGA repository’s sample sheet and manifest file. Additionally, the pipeline supports large-scale workflows and integrates well with broader, multi-language pipelines, making it more accessible and scalable for diverse users.

While the pipeline offers a solid foundation for further analyses, including multi-omics integration, and noticeably simplifies the process, there are some limitations. Currently, the script cannot handle all files due to varying naming conventions, although some exceptions have been incorporated. Future versions should aim to expand the number of exceptions, potentially with input from the community, to improve its versatility. Additionally, the lack of visualization for the download process is a minor limitation that could be addressed in future updates to enhance user experience. Currently, the TCGADownloadHelper is operating on unix-based systems. However, cross-platform compatibility can, to some extent, be achieved through the use of Snakemake, which is supported on Windows via conda. This provides a viable workaround for users operating in non-Unix environments, especially when combined with the pre-configured conda environments included in our repository. Nevertheless, for users unfamiliar with such setups, we are also exploring containerized solutions (e.g., Docker) to simplify deployment and ensure platform-independent reproducibility in future releases. Moving forward, further integration of our pipeline into platforms like cBioPortal could provide greater utility by streamlining data accessibility and visualization. This would be especially beneficial for researchers working with large-scale cancer datasets. Our pipeline is primarily designed for data preprocessing, including steps, such as data download and file renaming. We have implemented a prototype sample analysis pipeline in Snakemake. This could also be adapted for other platforms, such as KNIME or Galaxy, depending on users’ needs. Additionally, extensions to the pipeline are highly encouraged, as incorporating new data types and features could enhance its versatility. Researchers are welcome to contribute these extensions to our existing GitHub repository after thorough validation, fostering a collaborative approach to improving the tool and expanding its applicability.

In summary, we have developed a pipeline that simplifies TCGA data handling and prepares these datasets for further analyses. Our step-by-step guide provides valuable assistance, particularly for first-time users, making TCGA data more accessible and easier to manage.

## Data Availability

The original contributions presented in the study are included in the article/Supplementary Material, further inquiries can be directed to the corresponding author.
