# Supplementary material for: TCGADownloadHelper: simplifying TCGA data extraction and preprocessing
Source: Front Genet. 2025 May 2;16:1569290. doi: 10.3389/fgene.2025.1569290 (PMC12081331; doi:10.3389/fgene.2025.1569290)
Supplement: Supplementary file 1 [file Table1.pdf]

Supplementary Material

1 SUPPLEMENTARY TABLES AND FIGURES

1.1 Tables

Table S1. Comparison of a traditional manual procedure versus the TCGADownloadHelper pipeline for TCGA data preprocessing.

**Workflow Step**	**Manual Procedure**	**TCGADownloadHelper Pipeline**
Data selection from GDC	Manual selection through the web interface	Manual (same)
Manifest and sample sheet handling	User must manually locate and organize files	Folder structure is auto-generated by the pipeline
File renaming (opaque IDs to case IDs)	Requires scripting or manual mapping from sample sheet	Automatically maps file IDs to case IDs using sample sheet
Folder structure organization	Manual creation and file sorting	Symbolic links and organized folders are created automatically
Filtering previously downloaded files	Requires manual inspection or scripting	Built-in filtering avoids re-downloading existing files
Integration of multimodal data per patient	Requires manual tracking and matching of data types	Naming conventions allow easy cross-referencing via case ID
Reusability and reproducibility	Workflow varies per user; hard to reproduce exactly	Highly reproducible via standardized config and Snakemake rules
Error risk (e.g., renaming, mislabeling)	High - manual steps are error-prone	Low - automation ensures consistent naming
Execution method	Multiple manual steps and scripts; low automation	One-touch execution via Snakemake or Jupyter Notebook
Extendability (e.g., downstream analysis)	Requires additional scripting	Easily extendable via modular Snakemake rules
